# Bilateral Testicular Torsion in Bilateral Polyorchidism—A Case Report

**DOI:** 10.1155/2024/3676050

**Published:** 2024-09-26

**Authors:** Breno Lanter Cardoso, Bruno Roberto da Silva Ferreira, Élcio Dias Silva, Anuar Ibrahim Mitre

**Affiliations:** ^1^ Hospital Federal Lagoa, Fortaleza, Ceará, Brazil; ^2^ Hospital Sirio-Libanes, São Paulo, Brazil; ^3^Rua Adma Jafet 50, cj. 44, São Paulo 01308-050, Brazil

## Abstract

A 17-year-old patient with acute testicular pain had no blood flow observed on scrotal ultrasound Doppler on either side, suggestive of bilateral torsion. The patient underwent immediate scrotal surgical exploration, and a numerical anomaly was observed during surgery—there were two testicles on each side, and all four exhibited torsion. After detorsion, circulation was recovered, and three of four testicles were kept. One dystrophic testis was removed.

## 1. Introduction

Polyorchidism is very rare, and approximately 200 cases have been reported previously [1]. In most cases, there is a supernumerary testicle, constituting triorchidism [2], and its diagnosis is often associated with the presence of inguinal hernia, cryptorchidism, torsion, or neoplasia. Acute testicular torsion is a urological emergency that may be associated with the incidental discovery of polyorchism [1], and the surgeon should ascertain the vitality of the gonads to determine the surgical procedure.

Polyorchidism occurs in 15% of cases of testicular torsion, and cases have already been described in the literature [1–3].

We report a case of bilateral polyorchidism involving two gonads in each hemiscrotum that constituted a rare entity of four testicles [3] associated with bilateral acute testicular torsion. The case was confirmed by Doppler sonographic examination, and we present the details of diagnosis, treatment, and follow-up.

## 2. Case Report

A 17-year-old patient sought emergency care due to acute scrotal pain of sudden onset and high intensity lasting 18 h that did not improve with the use of analgesic and nonsteroidal anti-inflammatory drugs.

**Figure 1:**
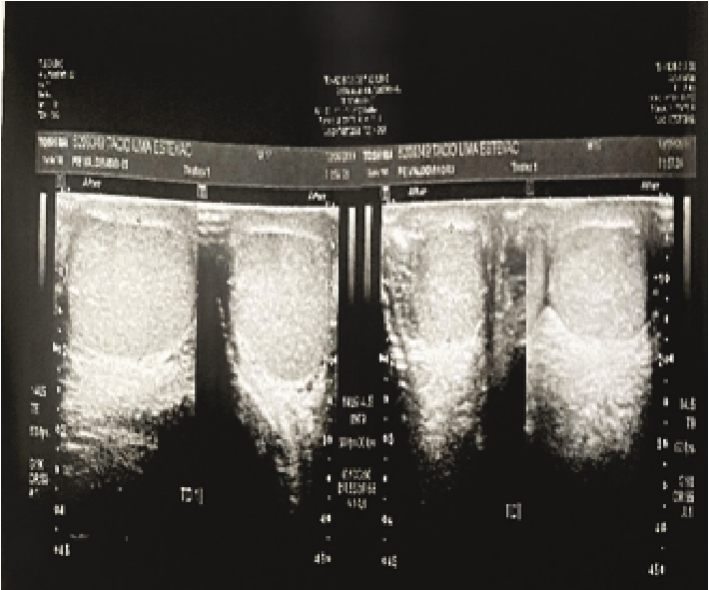
Ultrasound image of the scrotum.

He was evaluated by a general surgeon who observed phlogistic signs in the scrotum and difficult palpation due to pain and associated edema. Scrotal ultrasound examination revealed a topical right testicle, with normal morphology, reduced dimensions of 2.6 × 2.5 × 1.9 cm and an approximate volume of 6.6 cc, a right epididymis of normal echogenicity, a topical left testicle with morphology and normal dimensions—3.3 × 3.1 × 2.6 cm—and an approximate volume of 13.9 cc, and a left epididymis with normal characteristics. There was no Doppler-indicated blood flow in either testicle, suggestive of bilateral torsion [4] (Figure 1).

**Figure 2:**
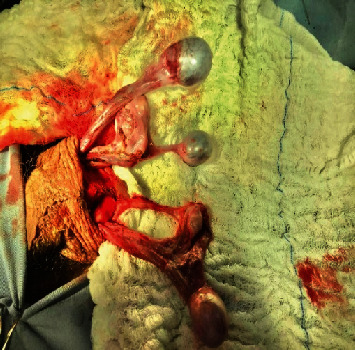
Surgical exploration showing numerical abnormalities on both sides. One of the testicles on the left side was atrophic.

The patient underwent scrotal surgical exploration, and a numerical anomaly was observed during surgery; on each side, there were two testicles, all of which were twisted counterclockwise along with an intact vaginal tunic and a single pedicle and epididymis. In the left hemiscrotum, one of the testicles was rudimentary and atrophic, noncommunicating with the larger testicle (Figure 2).

**Figure 3:**
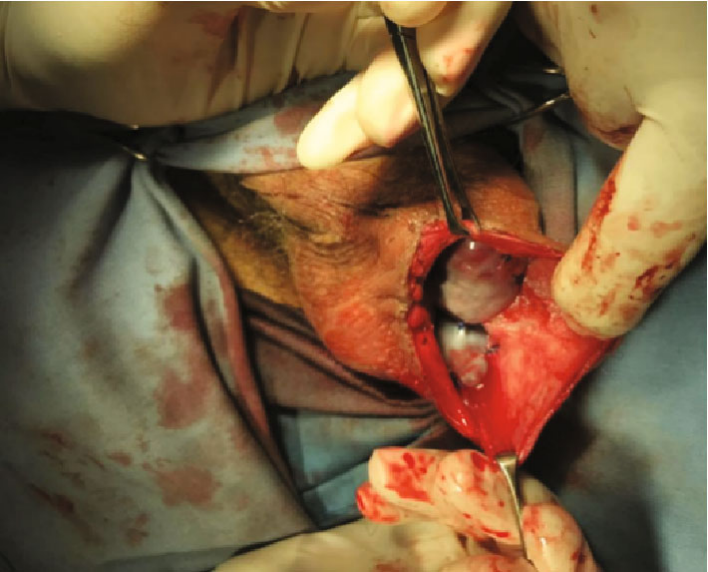
Parietal attachment of the testicles and completion of the procedure.

The detorsion of the testicles was carried out bilaterally, and the atrophic testicle was removed. After recovering blood circulation and evident improvement of appearance, the testicles were preserved and fixed in the scrotum (Figure 3).

The patient had a good postoperative recovery and was discharged from the hospital the following day. The pathological examination of the surgical specimen was compatible with testicular atrophy, revealing testicular parenchyma and stromal edema in the midst of immature seminiferous tubules and signs of marked ischemia.

Doppler scrotal ultrasound examination performed 10 days after the procedure revealed two normal right-sided testicles as well as one on the left, all morphologically complete and with blood circulation.

Four years later, the patient returned asymptomatic and satisfied with the aesthetic aspect of the scrotum. An echography study with Doppler was requested, which revealed polyorchidism in the right bag with good Doppler flow and maintained dimensions; the gonads were normal on physical and ultrasound examination.

**Figure 4:**
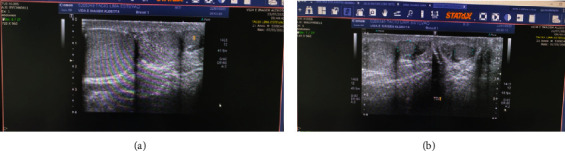
Ultrasound of the right hemiscrotum (a, b).

**Figure 5:**
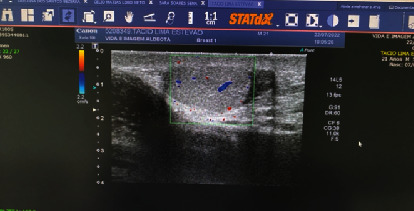
Ultrasound of the left hemiscrotum.

The sonographic documentation is shown in Figures 4 and 5.

## 3. Discussion

Polyorchidism is a rare condition, being documented in approximately 200 cases in the literature [1] and usually evidenced as a secondary event or incidental finding in research on cryptorchidism, torsion of the testicle, scrotal mass, and/or inguinal hernia in younger children [2].

The finding varies greatly in relation to the age of diagnosis. There are reports the condition being discovered in young children as well as in elderly patients [2].

The most common event is triorchidism associated with inguinal hernia, testicular torsion, or cryptorchidism, which should still always be observed for a higher risk of malignancy in the supernumerary testicle [2].

When discovered incidentally, fixation of the supernumerary testicle should be performed to avoid twisting, with the same orientation as adopted for the correction of inguinal hernia. If the position is fixed in a way that favors physical and imaging examination and avoids the risk of torsion, proper monitoring of the supernumerary organ in accordance with its higher risk of malignancy is facilitated [5].

Imaging diagnosis is generally made by ultrasound, usually performed for diagnostic investigation of other routine conditions such as definition of inguinal hernia, hydrocele, varicocele, or during urgent medical care in clinical suspicion of testicular torsion. The study by magnetic resonance imaging is more expensive and difficult to perform, especially in younger children and complicated cases of polyorchidism [6].

Ultrasound findings as well as magnetic resonance imaging findings are similar for the supernumerary testicle and the primordial testicle [4].

The extra testicles are usually intrascrotal in 75% of cases, with the left side being more commonly affected; in only 25% of the reports have they been located on the right side, and approximately 5% have been intra-abdominal. Most cases of polyorchidism are discovered during the investigation of inguinal hernia or cryptorchidism. Most of them are asymptomatic, and there are few cases of testicular torsion in supernumerary testicles [6].

The location, image, and characteristics of the supernumerary testicles guide treatment, accompanied by the knowledge that ectopic tissue has a greater possibility of malignant transformation based on its origin [1, 6].

There are doubts about the best surgical approach for polyorchidism. Consideration should be given to decision-making regarding the location of the supernumerary organ, morphology and its relationship to the testicle and its attachments, and the age of the patient and type of correlated condition. This is important because of possible future infertility if there is associated torsion [6] and possible malignant transformation if the organ is mainly ectopic [3].

Bilateral testicular torsion was found in the case described, and bilateral polyorchidism was observed during scrotal surgery. In this case, there were four testicles, which, to our knowledge, is a first.

By the characteristic of the testicles found in the right hemiscrotum, both were maintained for potential collaboration in reproductive and hormonal function, based on the improved aspects regarding vitality and the presence of a single spermatic cord and epididymis [7, 8].

In this case involving supernumerary testicles in a topical position, the improvement of the aspect of the testicles after detorsion enabled their preservation and fixation, resulting in a good progression both according to physical examination and by late documentation with scrotal Doppler ultrasound [7, 8].

## 4. Conclusion

We have presented a first-ever report of polyorchism with four testicles that were all twisted. Our intervention enabled the preservation of three of the four testicles.

## Data Availability

The authors declare that all data referring to the case reported are contained in the medical record at the Federal Hospital of Lagoa, Fortaleza, Ceará, Brazil, particularly, at the hospital's medical file service.
